# OptMDFpathway: Identification of metabolic pathways with maximal thermodynamic driving force and its application for analyzing the endogenous CO_2_ fixation potential of *Escherichia coli*

**DOI:** 10.1371/journal.pcbi.1006492

**Published:** 2018-09-24

**Authors:** Oliver Hädicke, Axel von Kamp, Timur Aydogan, Steffen Klamt

**Affiliations:** Max Planck Institute for Dynamics of Complex Technical Systems, Magdeburg, Germany; University of Washington, UNITED STATES

## Abstract

Constraint-based modeling techniques have become a standard tool for the *in silico* analysis of metabolic networks. To further improve their accuracy, recent methodological developments focused on integration of thermodynamic information in metabolic models to assess the feasibility of flux distributions by thermodynamic driving forces. Here we present *OptMDFpathway*, a method that extends the recently proposed framework of Max-min Driving Force (MDF) for thermodynamic pathway analysis. Given a metabolic network model, *OptMDFpathway* identifies both the optimal MDF for a desired phenotypic behavior as well as the respective pathway itself that supports the optimal driving force. *OptMDFpathway* is formulated as a mixed-integer linear program and is applicable to genome-scale metabolic networks. As an important theoretical result, we also show that there exists always at least one elementary mode in the network that reaches the maximal MDF. We employed our new approach to systematically identify all substrate-product combinations in *Escherichia coli* where product synthesis allows for concomitant net CO_2_ assimilation via thermodynamically feasible pathways. Although biomass synthesis cannot be coupled to net CO_2_ fixation in *E*. *coli* we found that as many as 145 of the 949 cytosolic carbon metabolites contained in the genome-scale model *i*JO1366 enable net CO_2_ incorporation along thermodynamically feasible pathways with glycerol as substrate and 34 with glucose. The most promising products in terms of carbon assimilation yield and thermodynamic driving forces are orotate, aspartate and the C4-metabolites of the tricarboxylic acid cycle. We also identified thermodynamic bottlenecks frequently limiting the maximal driving force of the CO_2_-fixing pathways. Our results indicate that heterotrophic organisms like *E*. *coli* hold a possibly underestimated potential for CO_2_ assimilation which may complement existing biotechnological approaches for capturing CO_2_. Furthermore, we envision that the developed *OptMDFpathway* approach can be used for many other applications within the framework of constrained-based modeling and for rational design of metabolic networks.

## Author summary

When analyzing metabolic networks, one often searches for metabolic pathways with certain (desired) properties, for example, conversion routes that maximize the yield of a product from a given substrate. While those problems can be solved with established methods of constraint-based modeling, no algorithm is currently available for genome-scale models to identify the pathway that has the highest possible thermodynamic driving force among all solutions with predefined stoichiometric properties. This gap is closed with our new approach *OptMDFpathway* which is based on the recently introduced concept of Max-min Driving Force (MDF). *OptMDFpathway* offers various applications, especially in the context of metabolic design of cell factories. To demonstrate the power and usefulness of *OptMDFpathway*, we employed it to analyze the endogenous CO_2_ fixation potential of *Escherichia coli*. While *E*. *coli* cannot assimilate CO_2_ into biomass, net CO_2_ fixation can take place along linear pathways from substrate to product and we show that thermodynamically feasible pathways with net CO_2_ assimilation exist for 145 (34) products when choosing glycerol (glucose) as substrate. Our results indicate that heterotrophic organisms like *E*. *coli* hold a possibly underestimated potential for CO_2_ assimilation which may complement existing biotechnological approaches for capturing CO_2_.

## Introduction

The reconstruction of organism-specific genome-scale metabolic models and their *in silico* analysis by techniques of constraint-based modeling has become a key to understand structure, function, and capabilities of metabolic networks [1–3]. Applications include the calculation of optimal flux distributions, e.g., with respect to growth or production of certain compounds (flux balance analysis (FBA), [1,4,5]), exploration of the space of feasible flux phenotypes by means of pathway vectors (e.g., via elementary flux modes [6–8] or elementary flux vectors [9]), prediction of reaction/gene essentialities, integration of different types of omics data, and the identification of optimal intervention targets for rational strain design [10].

Recently, more and more efforts have been made to integrate thermodynamic information into constraint-based analysis methods [11–30], especially into FBA-based approaches [12,17–21,25–27] and pathway-based techniques [13,15,16,28]. Two particular methods that have received much attention are thermodynamic FBA [17,26] and the Max-min driving force approach [29]. For thermodynamic FBA, additional variables for the Gibbs free energy change of the reactions together with constraints on metabolite concentrations are included in the optimization problem to identify optimal flux vectors (and a corresponding metabolite concentration vector) where all reactions proceed in the thermodynamically feasible direction. In contrast, the Max-min Driving Force (MDF) approach was proposed to determine optimal (maximal) thermodynamic driving forces for a given metabolic pathway [29]. If a pathway has a high MDF, then a metabolite concentration vector can be found where all participating reactions of the pathway have simultaneously high driving forces facilitating a high flux and/or a low enzyme requirement. Conversely, pathways with low MDF values will either have low flux or must be catalyzed by highly abundant enzymes to enable a significant flux. The MDF method was used to thoroughly analyze the thermodynamic efficiency of different pathways of the central metabolism [29] and to evaluate the potential of pathway designs for synthetic photo-electro-autotrophy [30]. Recently, the MDF method together with a pathway identification procedure was used to identify thermodynamically feasible synthetic pathways that were assembled with enzymes from different organisms [11]. However, the latter as well as the original approach for MDF computation presented in [29] necessitate that a specific pathway is given *a priori*. So far, no method exists that can directly identify pathways in a metabolic network with maximal MDF. One approach could be to enumerate the complete set of elementary modes (or other pathway vectors) followed by a subsequent computation of their respective MDF values. However, this approach is limited to medium-size network and can thus not be used for genome-scale models.

For this reason we formulate herein a mixed integer linear program (MILP) problem that optimizes the MDF with respect to different constraints (e.g. concentration ranges, ratio constraints, yield constraints, etc.) without the prerequisite to define a specific reaction sequence *a priori*. This MILP identifies the optimal MDF value together with a corresponding pathway (represented as a steady-state flux distribution). Hence, the result of *OptMDFpathway* is not only the optimal MDF value but also the associated pathway enabling the optimal driving force.

In the second part of this work, we employ our new *OptMDFpathway* approach to assess the endogenous potential of *Escherichia coli* to fix CO_2_ via thermodynamically feasible pathways. The development of sustainable bioprocesses using CO_2_ as feedstock to produce valuable chemicals and fuels from CO_2_ is highly desirable as they have several advantages compared to chemical CO_2_ reduction [31]. For example, only mild reaction conditions are required or low-purity reactants can be used. Many autotrophic microorganisms are capable of catalyzing the reduction of CO_2_ at ambient conditions. They incorporate CO_2_ into valuable organic compounds via six naturally occurring carbon fixation pathways [32]. Many of these organisms exhibit a phototrophic lifestyle where the Calvin-Benson-Bassham cycle [33] is the most abundant CO_2_ assimilation pathway. However, volumetric productivities and CO_2_ capturing rates are relatively small for phototrophic conditions since the maximal rate of the carboxylating enzyme Rubisco is an order of magnitude lower than the average of central metabolism enzymes [34] and efforts to improve Rubisco’s kinetic parameters were not sufficiently successful so far [35,36]. Also the necessary provision of suitable photobioreactors increases the costs of large-scale bioprocesses based on phototrophic organisms. Hence, there is still a need for faster and more efficient bioprocesses for the conversion of CO_2_ into valuable products. Recent research with respect to biotechnological potential for CO_2_ fixation includes the design of synthetic CO_2_ capturing cycles like the CETCH-cycle [37]. Studies on CO_2_ fixation in heterotrophic organisms have also been reported recently. For example, the Calvin-Benson-Bassham cycle has been incorporated to *Escherichia coli* to enable the synthesis of biomass components from CO_2_ [38], Generally, heterotrophic organisms have the advantage that growth and production rates are usually superior compared to the autotrophic life style.

Although wild-type *E*. *coli* cannot capture CO_2_ for pure biomass synthesis due to limited energy and redox supply by typical carbon substrates, a net assimilation of CO_2_ can take place when certain products are synthesized from a given substrate. For example, yield-optimal production of succinate with *E*. *coli* using glucose as substrate could result in synthesis of 1.71 mol succinate for each mol of glucose consumed. This corresponds to a conversion of 6 mol carbon from glucose and 0.86 mol carbon from CO_2_ into 6.86 mol carbon of succinate. In fact, for any succinate yield higher than 1.5 mol succinate per mol glucose, a net assimilation of CO_2_ takes place.

**Fig 1:**
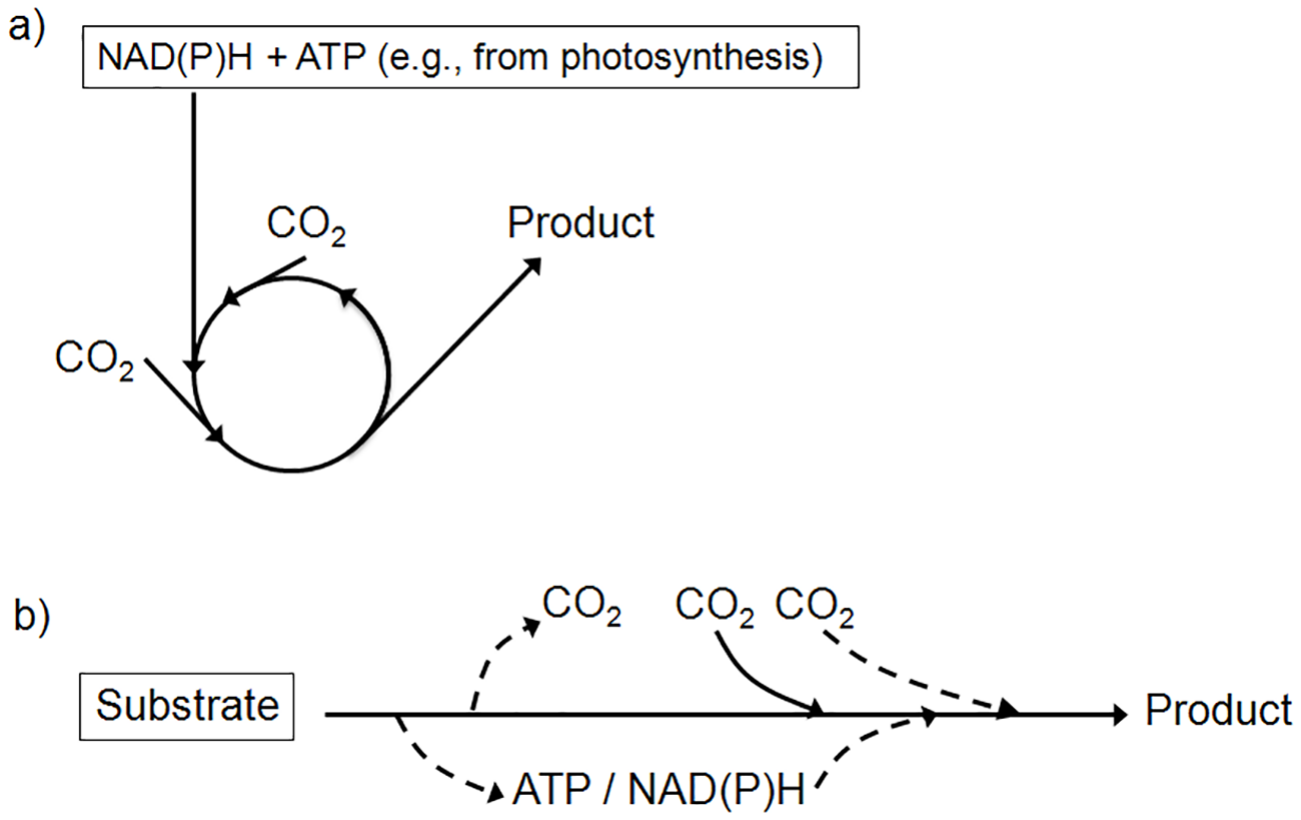
Autotrophic and heterotrophic CO_2_ fixation. a) Example of a typical autotrophic CO_2_ assimilation cycle (e.g. the Calvin-Benson-Bassham or reductive tricarboxylic acid cycle). ATP and reduction equivalents are provided by photo- or chemosynthesis. b) Heterotrophic CO_2_ assimilation can occur via linear pathways from a carbon substrate to certain products. If needed, ATP and/or reduction equivalents are generated from the substrate itself. Depending on the particular substrate-product combination, different amounts of CO_2_ can be assimilated.

In the present study we will systematically analyze the endogenous potential of *E*. *coli* to assimilate CO_2_ heterotrophically with two common substrates, glucose and glycerol. In contrast to classical cycles and pathways of CO_2_ fixation in autotrophic organisms, these pathways will typically represent linear pathways from the substrate to the respective product involving carboxylating reaction steps (Fig 1). We analyze both a core model for the central metabolism (EColiCore2 [39]) as well as a genome-scale model (*i*JO1366, [40]) and identify all substrate-product combinations with CO_2_ capturing potential in these models. Since CO_2_ fixation often requires overcoming high thermodynamic barriers, this kind of analysis essentially needs a method to search for pathways that are not only stoichiometrically but also thermodynamically feasible. The new *OptMDFpathway* method will enable us to identify genome-scale CO_2_ fixation pathways with reasonable driving force.

## Methods

### Max-min Driving Force (MDF)

For assessing the thermodynamic feasibility of a metabolic pathway Noor et al. introduced the concept of the Max-min driving force (MDF) [29]. The MDF provides an upper bound for the maximal thermodynamic driving force of a given pathway. The MDF approach requires as inputs a reaction sequence (the pathway) together with ranges for metabolite concentrations, the standard change in Gibbs energy Δ_*r*_*G*′^*o*^ for the participating reactions and (optionally) ratio constraints for some metabolite concentrations. The driving force of a single reaction is defined as the negative Gibbs free energy change of this reaction (−Δ_*r*_*G*′) and a reaction is thermodynamically feasible if this value is positive. The driving force of a pathway can in turn be defined as the minimum of all driving forces of the involved reactions (and a pathway is feasible if this minimum is positive). Hence, to maximize the driving force of a pathway, an optimization problem is formulated to identify a metabolite concentration profile that maximizes the minimum of all single reaction driving forces. In mathematical terms, this can be stated as a linear optimization problem [29]:

$$\begin{array}{c} \underset{\begin{array}{c} x,B \end{array}}{Maximize}\;B\\ Subject\;to-(\Delta_{r}G\prime^{o}+RT\cdot N^{T}x)\geq B\\ \text{ln}\left(C_{min}\right)\leq x\leq \text{ln}(C_{max}) \end{array}$$ (1)

*B* represents the lower bound for the driving force of all reactions participating in a given pathway which is maximized thus eventually yielding the MDF (in kJ/mol). Δ_*r*_***G***′^*o*^ is a vector containing the standard change in Gibbs energy of the involved reactions, ***N*** is the stoichiometric matrix (which includes the external metabolites), ***C***_***min***_ and ***C***_***max***_ are the vectors of metabolite concentration limits, ***x*** is the vector of logarithmized metabolite concentrations and *RT* is the product of the universal gas constant with temperature in Kelvin.

### Identification of MDF-optimal pathways via elementary modes

The original MDF approach presented in [29] requires a predefined pathway (or set of active reactions) as input. In this work we deal with finding pathways (for a given metabolic function) with maximal MDF. Hence, the optimal pathway is not known beforehand and needs to be identified together with its (optimal) MDF value. In core or medium-scale networks, this can be done as follows: one enumerates all elementary (flux) modes (EMs) [6–8], computes for each relevant EM (e.g., exceeding a given minimum product yield) its MDF and finally ranks these EMs with respect to their MDF value thus yielding the pathway with maximum driving force at the top of this list. This approach is exhaustive and using EMs as pathways brings the advantage that all pathways are balanced with respect to their intermediate metabolites.

### OptMDFpathway: Identification of MDF-optimal pathways in genome-scale networks

Complete EM enumeration is normally not possible in genome-scale metabolic networks due to the combinatorial explosion of possible pathways. Again, we cannot simply use the entire network as input for finding a thermodynamically feasible pathway with maximum MDF since in problem (1) it is demanded that *all* reactions of the network are thermodynamically feasible whereas only a subset of all reactions in the network will be active in the optimal pathway. What is needed here is a method that identifies, for a desired phenotypic behavior, both the optimal MDF and a pathway that enables this MDF.

Therefore, we formulate *OptMDFpathway*, a mixed-integer linear program (MILP) that is applicable to genome-scale networks and identifies, for a specified (desired) phenotype, balanced (steady-state) flux distributions that are optimal with respect to their MDF. This MILP combines the original MDF optimization problem (1) with standard constraints used in flux balance analysis (FBA, [5]) and ensures that the reactions *which are active* in the solution (non-zero reaction rate) are all thermodynamically feasible (i.e., their driving force is greater than zero for the direction in which it operates).

The basis of the MILP is formed by the following equations:

$$\hat{N}r=0$$ (2)

$$\alpha_{i}\leq r_{i}\leq \beta_{i}$$ (3)

Dr≤d (4)

The vector ***r*** contains the (net) reaction rates. In contrast to the stoichiometric matrix ***N*** used in Eq (1), the matrix $\hat{N}$ comprises the internal metabolites only and can be obtained by removing the rows corresponding to external metabolites from ***N***. Constraints (2) and (3) are the same as in standard FBA problems (steady-state assumptions, flux bounds *α*_*i*_ and *β*_*i*_ which include non-negativity constraints for irreversible reactions) while (4) can optionally be used to add other inequality constraints (like yield constraints) to specify (desired) phenotypes. We again quantify the driving forces of reactions as their negative change of Gibbs free energy (cf. Eq (1)) and collect them in vector ***f***:

$$f_{i}=-\Delta_{r}G_{i}^{\prime }=-(\Delta_{r}G_{i}^{\prime o}+RT\cdot N_{∙,i}^{T}\cdot x).$$ (5)

where $N_{∙,i}^{T}$ is the transposed *i*-th column (reaction) of the (full) stoichiometric matrix ***N*** (as in Eq (1), ***N*** includes the external metabolites). Again, the logarithmized metabolite concentrations in ***x*** must thereby comply with the given concentration ranges:

$$\text{ln}\left(C_{min}\right)\leq x\leq \text{ln}(C_{max}).$$ (6)

In a preprocessing step we determine the minimum (*f*_*i*,*min*_) and maximum (*f*_*i*,*max*_) values for each driving force *f*_*i*_ subject to the concentration ranges (6).

Each reaction is associated with a binary variable *z*_*i*_ that must be 1 when a flux flows through this reaction. This is achieved by the constraint

$$r_{i}\leq z_{i}\cdot \beta_{i}$$ (7)

In order for this to work it is necessary to split the reversible reactions into the forward and reverse directions and to adjust the flux bounds (3) of the separate directions accordingly. The opposite directions of a given reversible reaction always have the same absolute driving force value but with opposite signs. For a given concentration vector ***x***, the direction with driving force greater than zero is the direction in which the net flux through that reaction flows.

In order to maximize the minimal driving force of all *active* reactions (the driving force of inactive reactions with zero flux is not taken into account) the following constraints are added to the optimization problem:

$$f_{i}\;+\left(1-z_{i}\right)\cdot M_{i}\geq B$$ (8)

With *K* = max(*f*_*i*,*max*_), we set *M*_*i*_ = *K* − *f*_*i*,*min*_ and because *B* ≤ *K* these constraints are always fulfilled for all reactions with *r*_*i*_ = *z*_*i*_ = 0. For all reactions with *z*_*i*_ = 1 it must hold that *f*_*i*_ ≥ *B*. Using the same objective function as in (1)

$$\underset{\begin{array}{c} x,r,B \end{array}}{Maximize}\;B$$ (9)

but this time subject to Eqs (2)–(8) (Eqs (3), (5), (7) and (8) for each reaction *i*), results in a mixed-integer linear program (MILP). Its solution (***x***, ***r***, *B*) will deliver a (not necessarily unique) flux distribution ***r*** where, given the calculated concentration vector ***x***, each active reaction has a driving force of at least *B* and there is no other steady-state flux distribution in the network with a higher *B* (MDF) with this property. In other words, the flux vector ***r*** found by the MILP represents a pathway with maximum MDF.

If concentration ratios of certain metabolites are assumed to be fixed (*c*_*i*_/*c*_*j*_ = *h*) then constraints of the form

$$x_{i}-x_{j}=\text{ln}\left(h\right)$$ (10)

can be added to the MILP.

### Metabolic models

For analyzing the endogenous CO_2_ fixation potential of *E*. *coli* we analyzed both a core as well as a genome-scale metabolic model. To get a comprehensive overview of all possible CO_2_ fixing pathways we used the *i*JO1366 model [40] which comprises 1805 metabolites and 2583 reactions. We studied in parallel a smaller core model of *E*. *coli*’s metabolism because it allows us (1) to fully enumerate and then assess all pathways (EMs) for net CO_2_ fixation and thereby to highlight the differences between the EM-based and *OptMDFpathway*-based approach for determining thermodynamically favorable pathways and (2) to focus only on well-known (major) pathways of the central metabolism thus excluding possibly unrealistic results due to pathways with low capacity in the genome-scale model. As core model of the central metabolism we used *EColiCore2* (ECC2) which is a sub-network of *i*JO1366 comprising 499 reactions and 486 metabolites [39,40]. ECC2 conserves major properties of *i*JO1366 but its moderate size allows for the complete enumeration of EMs.

As substrates we considered glucose and glycerol. Substrate uptake fluxes were normalized to 1 mmol/gCDW/h in both models and no further flux bounds were used. To avoid that succinate is a mandatory byproduct under anaerobic conditions (which is in conflict with experimental findings) we allowed reaction R_nadh16tpp (NADH dehydrogenase) to be reversible (as in ECC2, see [39]). Whenever we consider an internal metabolite as a potential product an auxiliary excretion reaction was temporarily added before the respective calculations for this product were performed.

### Calculation of Δ_*r*_*G*′^*o*^ and assumed metabolite concentration limits

Standard Gibbs free energy changes Δ_*r*_*G*′^*o*^ were determined for all cytosolic reactions where either a mapping for model reaction names to KEGG-IDs [41] was available in the BIGG database [42] or where a mapping for all metabolite names to KEGG compound IDs was possible. The Δ_*r*_*G*′^*o*^ were calculated via the component contribution method [43,44] for pH 7.0 and ionic strength of 0.1 by means of the eQuilibrator database and related script files [45]. Δ_*r*_*G*′^*o*^ (with associated uncertainties) could be determined for 298 and 691 out of the 397 and 1272 cytosolic reactions contained in ECC2 and *i*JO1366, respectively. Generally, Δ_*r*_*G*′^*o*^ was not considered for transport reactions in both ECC2 and *i*JO1366 because these values depend strongly on environmental conditions in the compartments like pH, membrane potential, ionic strength and concentration ranges of external metabolites.

The concentration limits for all metabolites were set to 1 μM as lower limit and 20 mM as upper limit. The concentration of CO_2_ was more strictly bounded to be in the range from 100 nM to 100 μM. The concentration ratios for ATP:ADP, ADP:AMP, NAD:NADH, NADPH:NADP and ${HCO}_{3}^{-}$:CO2 were fixed to 10:1, 1:1, 10:1, 10:1 and 2:1, respectively.

### Implementation and network calculations

The *OptMDFpathway* algorithm was implemented as a new function in the MATLAB toolbox *CellNetAnalyzer* [46] and all calculations (including EMs as well as flux balance and flux variability analyses) were performed with MATLAB scripts using API functions of *CellNetAnalyzer* [47,48].

## Results

### Reactions using CO_2_ or bicarbonate as substrates

In a first step we identified all CO_2_ or bicarbonate (${HCO}_{3}^{-}$) capturing reactions in both the core and the genome-scale model (Table 1). We found nine such reactions in *i*JO1366 (Table 1). Carbonic anhydrase is listed as one of those, but in the following we will not consider this reaction as a carbon capturing reaction as it only supports the conversion of CO_2_ into bicarbonate. From their stoichiometry and reversibility, the other eight reactions hold the potential to truly fix CO_2_ or ${HCO}_{3}^{-}$ in *i*JO1366; six of them are irreversible reactions while the backward flux of two reversible reactions (isocitrate dehydrogenase (R_ICDHy) and pyruvate synthase (R_POR5)) by definition allows for carbon incorporation (Table 1).

**Table 1:** CO_2_ and ${HCO}_{3}^{-}$ capturing reactions in the genome-scale model *i*JO1366. The last column indicates which of these reactions are contained in ECC2.

Reaction name	Enzyme	Stoichiometry	Driving force range	ECC2
R_PPC	PEP carboxylase	M_co2_c + M_h2o_c + M_pep_c ⇒	[-34.1–71.9]	✓
		M_h_c + M_oaa_c + M_pi_c		
R_CBPS	Carbamoyl phosphate synthase	2 M_atp_c+ M_gln_L_c + M_h2o_c + M_hco3_c ⇒ 2 M_adp_c + M_cbp_c + M_glu_L_c + 2 M_h_c + M_pi_c	[-26.7–95.0]	X (✓)
R_AIRC2	Phosphoribosylamino-imidazole carboxylase	M_air_c + M_atp_c + M_hco3_c ⇒	[7.1–117.1]	✓
		M_5caiz_c + M_adp_c + M_h_c + M_pi_c		
R_ACCOAC	Acetyl-CoA carboxylase	M_accoa_c + M_atp_c + M_hco3_c ⇒	[-41.7–49.1]	X
		M_adp_c + M_h_c + M_malcoa_c + M_pi_c		
R_DBTS	Dethiobiotin synthase	M_atp_c + M_co2_c + M_dann_c ⇒	[-73.3–32.5]	X
		M_adp_c + M_dtbt_c + 3 M_h_c + M_pi_c		
R_HCO3E	Carbonic anhydrase	M_co2_c + M_h2o_c ⇒	[-6.9–9.9]	✓
		M_h_c + M_hco3_c		
R_CBMKr	Carbamate kinase	1 M_atp_c + 1 M_co2_c + 1 M_nh4_c ⇒	[-89.0– -11.2]	✓ (X)
		1 M_adp_c + 1 M_cbp_c + 2 M_h_c		
R_POR5	Pyruvate synthase	M_coa_c + 2 M_flxso_c + M_pyr_c ⇔	[-74.6–147.8]	X
		M_accoa_c + M_co2_c + 2 M_flxr_c + M_h_c		
R_ICDHyr	Isocitrate dehydrogenase	M_icit_c + M_nadp_c ⇔	[-13.0–64.5]	✓
		M_akg_c + M_co2_c + M_nadph_c		

By minimizing and maximizing the thermodynamic driving force separately for each of these reactions (with the metabolite concentration ranges given in the Methods), we found that, in isolation, all of the above reactions except the carbamate kinase reaction (R_CBMKr) hold the thermodynamic potential to proceed in direction of carboxylation since the upper (lower) limits of the driving forces for the irreversible (reversible) reactions are positive (negative). The carbamate kinase reaction (R_CBMKr), which catalyses the first step in the urea cycle, is defined in *i*JO1366 such that it consumes only one mol of ATP and ammonia for synthesis of carbamoyl phosphate. However, the overall reaction is known to proceed via three separate chemical reactions where two moles of ATP for the synthesis of one molecule of carbamoyl phosphate are utilized and the relevant nitrogen substrate under physiological conditions is glutamine [49–51]. Because of the questionable stoichiometry and the thermodynamic infeasibility we neglected R_CBMKr form further analyses with *i*JO1366, however, carbamoyl phosphate can still be produced by the reaction of the carbamate kinase (R_CBPS, Table 1). In ECC2, which contained originally only reaction R_CBMKr, we replaced the latter by R_CBPS.

Further, although the backward flux of the endogenous pyruvate synthase (R_POR5) reaction in the genome-scale model of *E*. *coli* is thermodynamically feasible, it remains questionable under relevant physiological conditions. It has been shown in other microorganisms that this reaction can proceed in direction of carboxylation [52,53], however, the corresponding cofactor for this functionality is normally ferredoxin [52]. Given that the cofactor of pyruvate synthase in *E*. *coli* is flavodoxin and not ferredoxin [54], the feasibility of a carboxylation function under physiological conditions remains highly unlikely as the redox potential of flavodoxin seems not sufficient to support CO_2_ reduction. Therefore, to avoid an unrealistic assumption, we set this reaction initially to irreversible and discuss the sensitivity of the results with respect to this modification afterwards. Hence, at this point, from the nine reactions shown in Table 1, only six reactions do contribute to the CO_2_ assimilation capabilities in *E*. *coli*. The core model ECC2 contains four of these six CO_2_ assimilating reactions (Table 1).

Next, we used flux variability analysis to check for each of the above reactions whether there exist stationary flux distributions with a positive flux of the respective reaction in direction of carboxylation. We could find such a flux distribution for all of these reactions except for the isocitrate dehydrogenase reaction (R_ICDHyr). Although the latter is thermodynamically feasible in both directions, there is no balanced flux distribution in either of the two models that carries a negative flux for this reaction and it can thus not be used for CO_2_ fixation.

At this point we can thus conclude that CO_2_ (or ${HCO}_{3}^{-})$ incorporation in *E*. *coli* is facilitated by one of the first five reactions given in Table 1. Metabolites whose synthesis enables CO_2_ assimilation should either be products of these reactions or be transformed into different compounds by subsequent reactions. However, it is still not clear whether and for which metabolites balanced flux distributions exist in the network that lead to *net* CO_2_ consumption.

### Stoichiometric carbon fixation potential

In a next step we therefore used classical flux balance analysis [1,5] (without thermodynamic constraints except reaction reversibility) to identify substrate-product combinations which allow net CO_2_ consumption. Since we are mainly interested in identifying intracellular pathways that allow for CO_2_ incorporation, we restrict our analyses of potential products to the set of cytosolic carbon metabolites. Periplasmic and extracellular metabolites (which occur in almost all cases also as cytosolic species in the model) were not considered as possible products because analyses of the thermodynamic properties for the corresponding pathways would strongly rely on assumed environmental conditions in the specific compartments like pH, membrane potential, ionic strength or feasible concentration ranges. In total, 380 cytosolic carbon metabolites of ECC2 and 949 metabolites of *i*JO1366 were considered as potential (end) products for CO_2_ assimilation.

For each considered potential product an auxiliary excretion reaction was temporarily added and a flux optimization (flux balance analysis; FBA) problem formulated with maximization of the respective excretion reaction as its objective. Since the substrate uptake rate is the only applied constraint, the resulting maximized excretion rates (normalized to the substrate uptake rate) coincide with optimal product yields [55]. We defined the *CO*_*2*_ *assimilation yield* $Y_{{CO}_{2}/C_{S}}$ (normalized to molar carbon content of the substrate) as

$$Y_{CO_{2}/C_{S}}=\;\frac{C_{P}*Y_{P/S}\;-\;C_{S}}{C_{S}}$$ (11)

with *C*_*P*_ and *C*_*S*_ representing the molar carbon content of the product and substrate and *Y*_*P*/*S*_ as the molar product yield. Substrate-product combinations with net CO_2_ assimilation were identified by selecting all products for which the CO_2_ assimilation yield is equal or greater than 0.01 which guarantees that at least 1% CO_2_ (with respect to molar carbon uptake) is assimilated in the particular product of interest. Equivalent measures for CO_2_ fixation in terms of yield are, for example, (i) the fixed CO_2_ per mol substrate $Y_{{CO}_{2}/S}=Y_{{CO}_{2}/C_{S}}*C_{S}$ or (ii) the carbon-normalized product yield $Y_{P/S}^{C-norm}=\frac{C_{P}}{C_{S}}*Y_{P/S}=Y_{{CO}_{2}/C_{S}}+1$.

In the core model ECC2, we found that synthesis of 62 of the 380 cytosolic carbon metabolites (16.1%) allows for concomitant CO_2_ assimilation when using glycerol as substrate (see Table 2 (top-ranked products) and S1 Table (all products)). Thereof, 18 can also be synthesized from glucose (Tables 2 and S2). The higher number of possible products when using glycerol as substrate can be explained by its higher degree of reduction (5.3) compared to glucose (4.0). Also, the carbon-normalized CO_2_ assimilation yields $Y_{{CO}_{2}/C_{S}}$ are always higher when using glycerol compared to those based on glucose (except for oxaloacetate where identical yields are observed). Well-known products with net CO_2_ fixation are the C4-metabolites of the reductive TCA cycle branch oxaloacetate, malate, fumarate and succinate as they are part of a linear reaction sequence following phosphoenolpyruvate carboxylase. These metabolites can be produced either with glucose or glycerol as substrate and allow for the assimilation of up to 0.33 mol CO_2_ per C-mol substrate metabolized (e.g. for oxaloacetate, Table 2). However, the best normalized CO_2_ fixation yield is possible with orotate produced from glycerol (Table 2). Its maximal product yield is 0.93 mol orotate per mol glycerol which corresponds to a molar carbon assimilation yield of 0.55 mol CO_2_ per C-mol glycerol. In other words, for each supplied mol of glycerol another 1.65 mol of CO_2_ are assimilated at yield-optimal conditions. In this case, CO_2_ accounts for 35.5% of all carbon atoms of the synthesized orotate. Less obvious products that can be synthesized with high CO_2_ assimilation yields with both substrates are e.g. aspartate and asparagine. One metabolite that allows CO_2_ fixation on glycerol but not on glucose is, for instance, homoserine.

**Table 2:** Maximal CO_2_ assimilation yields and thermodynamic properties for the top 15 products in the core model ECC2 with glucose or glycerol as substrate. #C-atoms: number of carbon atoms per product.$\;Y_{{CO}_{2}/S}^{max}$: maximal yield of fixed CO_2_ per mol substrate consumed; $Y_{{CO}_{2}/C_{S}}^{max}$: maximal (carbon-normalized) CO_2_ assimilation yield; $Y_{P/S}^{max}\;(MDF)$: maximal product yield (corresponding optimal MDF in parentheses). $Y_{P/S}^{max}\;at\;MDF\geq 3.0$: maximal product yield if a minimal MDF of 3.0 is demanded (minimal pathway length with MDF ≥ 3.0 in parentheses). *MDF*_*max*_ ($Y_{{CO}_{2}/C_{S}}^{max}$): maximal MDF (corresponding maximal CO_2_ assimilation yield in parentheses).

Product	#C-atoms	$Y_{{CO}_{2}/S}^{max}\;/\;Y_{{CO}_{2}/C_{S}}^{max}\;/\;Y_{P/S}^{max}$ (MDF)	$Y_{P/S}^{max}\;at\;MDF\geq 3.0$ (pathway length)	*MDF*_*max*_ ($Y_{{CO}_{2}/C_{S}}^{max})$
****Glucose****				
Oxaloacetate	4	1.98 / 0.33 / 2.00 (7.1)	2.00 (12)	8.6 (0.17)
Orotate	5	1.92 / 0.32 / 1.59 (0.3)	1.57 (26)	8.6 (0.08)
Iminoaspartate	4	1.80 / 0.30 / 1.95 (3.3)	1.95 (19)	8.6 (0.07)
Fumarate	4	1.62 / 0.27 / 1.90 (3.3)	1.90 (16)	8.6 (0.04)
L-Malate	4	1.62 / 0.27 / 1.90 (3.3)	1.90 (15)	8.6 (0.04)
(S)-Dihydroorotate	5	1.44 / 0.24 / 1.49 (0.3)	1.46 (29)	8.6 (0.01)
L-Aspartate	4	1.38 / 0.23 / 1.84 (3.3)	1.84 (17)	8.6 (0.01)
N-Carb-L-aspartate	5	1.32 / 0.22 / 1.47 (0.3)	1.44 (28)	7.1 (0.15)
4-P-L-Aspartate	4	0.90 / 0.15 / 1.73 (3.3)	1.73 (24)	7.1 (0.08)
L-Asparagine	4	0.90 / 0.15 / 1.73 (-3.0)	--	-1.0 (0.08)
Succinate	4	0.84 / 0.14 / 1.71 (3.3)	1.71 (21)	7.1 (0.10)
Carbamoyl phosphate	1	0.72 / 0.12 / 6.71 (0.3)	6.07 (27)	3.3 (0.01)
Quinolinate	7	0.60 / 0.10 / 0.95 (7.1)	0.95 (22)	8.6 (0.01)
Dihydrodipicolinate	7	0.60 / 0.10 / 0.94 (3.3)	0.94 (25)	7.1 (0.07)
Orotidine-5-P	10	0.42 / 0.07 / 0.64 (-3.0)	--	-1.0 (0.03)
****Glycerol****				
Orotate	5	1.65 / 0.55 / 0.93 (-7.8)	0.87 (23)	8.6 (0.15)
(S)-Dihydroorotate	5	1.35 / 0.45 / 0.87 (-7.8)	0.82 (23)	8.6 (0.08)
N-Carb-L-aspartate	5	1.29 / 0.43 / 0.86 (-7.8)	0.80 (22)	8.6 (0.06)
L-Aspartate	4	0.99 / 0.33 / 1.00 (4.5)	1.00 (12)	8.6 (0.08)
Fumarate	4	1.00 / 0.33 / 1.00 (4.5)	1.00 (11)	8.6 (0.10)
Iminoaspartate	4	1.00 / 0.33 / 1.00 (5.2)	1.00 (14)	8.6 (0.14)
L-Malate	4	1.00 / 0.33 / 1.00 (4.5)	1.00 (10)	8.6 (0.10)
Oxaloacetate	4	1.00 / 0.33 / 1.00 (7.5)	1.00 (10)	8.6 (0.24)
Succinate	4	1.00 / 0.33 / 1.00 (2.6)	0.96 (16)	8.6 (0.03)
4-P-L-Aspartate	4	1.00 / 0.33 / 1.00 (-7.8)	0.94 (17)	7.5 (0.02)
L-Asparagine	4	1.00 / 0.33 / 1.00 (-9.7)	0.88 (21)	7.5 (0.02)
Carbamoyl phosphate	1	0.87 / 0.29 / 3.86 (0.3)	3.50 (18)	5.2 (0.17)
Orotidine-5-P	10	0.72 / 0.24 / 0.37 (-9.7)	0.33 (31)	7.7 (0.03)
Oxoheptanedioate	7	0.66 / 0.22 / 0.33 (-7.8)	0.31 (27)	5.2 (0.10)
L-Asp-semialdehyde	4	0.60 / 0.20 / 0.90 (-7.8)	0.84 (24)	5.2 (0.05)

In *i*JO1366, as many as 253 metabolites (26.7% of all 949 cytosolic carbon metabolites) can be synthesized with net CO_2_ fixation with glycerol as substrate (Tables 3 and S3). Thereof, 41 can also be synthesized from glucose with concomitant CO_2_ fixation (Tables 3 and S4). Despite the much larger number of metabolites whose synthesis allows in principle for CO_2_ assimilation, the ranking of top candidates in *i*JO1366 is very similar to ECC2 (Table 3). The C4-metabolites oxaloacetate, orotate, and aspartate of the reductive TCA cycle branch are again the products with highest carbon assimilation yields. However, for some products, the maximum carbon assimilation yields are up to 10% higher compared to ECC2 indicating that some pathways contained in *i*JO1366 but not in ECC2 allow even higher CO_2_ assimilation. Also, some new products show up as promising candidates, for example, (iso)citrate.

**Table 3:** Maximal CO_2_ assimilation yields and thermodynamic properties for the top 15 products in the genome-scale model *i*JO1366 with glucose or glycerol as substrate. #C-atoms: number carbon atoms per product.$\;Y_{{CO}_{2}/S}^{max}$: maximal yield of fixed CO_2_ per mol substrate; $Y_{{CO}_{2}/C_{S}}^{max}$: maximal (carbon-normalized) CO_2_ assimilation yield; $Y_{P/S}^{max}\;(MDF)$: maximal product yield (corresponding maximal MDF in parentheses). $Y_{P/S}^{max}\;$*at MDF* ≥ 3.0: maximal product yield if a minimal MDF of 3.0 is demanded (minimal pathway length with *MDF* ≥ 3.0 in parentheses). *MDF*_*max*_ ($Y_{{CO}_{2}/C_{S}}^{max}$): maximal MDF (with maximal CO_2_ assimilation yield in parentheses).

Product	#C-atoms	$Y_{{CO}_{2}/S}^{max}\;/\;Y_{{CO}_{2}/C_{S}}^{max}\;/\;Y_{P/S}^{max}$ (MDF)	$Y_{P/S}^{max}\;at\;MDF\geq 3.0$ (pathway length)	*MDF*_*max*_ ($Y_{{CO}_{2}/C_{S}}^{max})$
****Glucose****				
Oxaloacetate	4	2.22 / 0.37 / 2.062 (0.3)	2.057 (12)	8.6 (0.17)
Iminoaspartate	4	2.10 / 0.35 / 2.02 (-37.9)	1.95 (16)	8.6 (0.07)
Orotate	5	1.92 / 0.32 / 1.59 (0.3)	1.58 (25)	8.6 (0.08)
Fumarate	4	1.62 / 0.27 / 1.90 (4.5)	1.90 (14)	8.6 (0.04)
L-Malate	4	1.62 / 0.27 / 1.90 (4.5)	1.90 (13)	8.6 (0.04)
(S)-Dihydroorotate	4	1.44 / 0.24 / 1.49 (0.3)	1.47 (28)	8.6 (0.01)
L-Aspartate	4	1.38 / 0.23 / 1.84 (4.5)	1.84 (17)	8.6 (0.01)
Aconitate/Citrate/IsoCitrate	6	1.08 / 0.18 / 1.185 (2.2)	1.182 (28)	8.6 (0.05)
Methylaconitate	7	1.02 / 0.17 / 1.00 (4.5)	1.00 (15)	8.6 (0.02)
4-P-L-aspartate	4	0.90 / 0.15 / 1.73 (4.5)	1.73 (22)	7.1 (0.08)
L-Asparagine	4	0.90 / 0.15 / 1.73 (-2.2)	1.60 (26)	7.1 (0.01)
Succinate	4	0.84 / 0.14 / 1.71 (4.5)	1.71 (20)	7.1 (0.10)
Quinolinate	7	0.78 / 0.13 / 0.97 (-37.9)	0.95 (21)	8.6 (0.01)
Carbamoyl phosphate	1	0.72 / 0.12 / 6.71 (0.3)	6.29 (25)	7.1 (0.02)
Glyoxylate	2	0.66 / 0.11 / 3.34 (0.3)	3.11 (37)	7.1 (0.01)
****Glycerol****				
Orotate	5	1.65 / 0.55 / 0.93 (-7.8)	0.91 (21)	8.6 (0.15)
Oxaloacetate	4	1.38 / 0.46 / 1.10 (-7.8)	1.08 (10)	8.6 (0.24)
(S)-Dihydroorotate	4	1.35 / 0.45 / 0.87 (-7.8)	0.86 (22)	8.6 (0.08)
Iminoaspartate	4	1.29 / 0.43 / 1.07 (-37.9)	1.04 (13)	8.6 (0.14)
Fumarate	4	1.17 / 0.39 / 1.04 (-7.8)	1.03 (11)	8.6 (0.10)
L-Malate	4	1.17 / 0.39 / 1.04 (-7.8)	1.03 (10)	8.6 (0.10)
L-Aspartate	4	1.14 / 0.38 / 1.03 (-7.8)	1.02 (12)	8.6 (0.08)
4-P-L-aspartate	4	1.02 / 0.34 / 1.01 (-7.8)	0.98 (16)	7.5 (0.07)
L-Asparagine	4	1.02 / 0.34 / 1.01 (-9.7)	0.92 (21)	7.5 (0.07)
Aconitate/Citrate/IsoCitrate	6	1.02 / 0.34 / 0.67 (-7.8)	0.65 (26)	8.6 (0.10)
Succinate	4	1.00 / 0.33 / 1.00 (4.3)	1.00 (14)	8.6 (0.03)
Carbamoyl phosphate	1	0.87 / 0.29 / 3.86 (0.3)	3.64 (18)	7.5 (0.02)
Methylaconitate	7	0.78 / 0.26 / 0.54 (-7.8)	0.53 (16)	8.6 (0.08)
Glyoxylate	2	0.75 / 0.25 / 1.88 (-7.8)	1.72 (35)	8.6 (0.02)
Orotidine-5-P	10	0.72 / 0.24 / 0.37 (-9.7)	0.34 (29)	8.3 (0.03)

We then used flux variability analysis in both models to determine which of the carbon assimilation reactions are essential for the identified substrate-product combinations (Table 4). The PEP carboxylase reaction (R_PPC) is most often essential followed by the reactions of the phosphoribosylamino-imidazole carboxylase (R_AIRC2) and carbamoyl phosphate synthase (R_CBPS) which are essential for a smaller number of products. The two reactions of the acetyl-CoA carboxylase (R_ACCOAC) and dethiobiotin synthase (R_DBTS) (exclusively) contained in *i*JO1366 are not essential for any substrate-product combination. These two reactions fix only minor amounts of CO_2_ when biomass components are produced and they cannot contribute to net CO_2_ fixation in any product. This implies that from the five reactions in *i*JO1366 (three reactions in ECC2) where CO_2_ or ${HCO}_{3}^{-}$ is defined as consumable reactant (Table 1), eventually only three reactions are accountable for *E*. *coli’s* carbon (net) fixation abilities.

**Table 4:** Number of products with net CO_2_ fixation for which a carboxylation reaction is essential and number of products requiring one or two essential carboxylation reactions.

Reaction	Glucose		Glycerol	
	ECC2	*i*JO1366	ECC2	*i*JO1366
R_PPC	17	35	40	191
R_CBPS	5	5	16	49
R_AIRC2	0	2	28	64
R_ACCOAC	X	0	X	0
R_DBTS	X	0	X	0
One / two essential carboxylation reactions	14 / 4	40 / 1	40 / 22	202 / 51
Total number of products with net CO_2_ fixation	18	41	62	253

We furthermore found that the production of each metabolite requires at least one specific essential carbon assimilation reaction(s) meaning that all alternative production pathways for each metabolite share the same essential carboxylation reaction(s) (Table 4). In the core model ECC2, for 22 of the 62 metabolites whose synthesis from glycerol allows for concomitant CO_2_ assimilation, the simultaneous activity of two carboxylation reactions in ECC2 is required whereas the remaining 40 require only one carboxylation reaction. With glucose as substrate, there are four products (N-carbamoyl-L-aspartate, (S)-dihydroorotate, orotate, orotidine-5-P) that require two carboxylation reactions. In *i*JO1366 with glucose, only one metabolite (orotidine-5-P) essentially requires two carboxylation reactions and 51 metabolites with glycerol, respectively. At yield optimality, the number of metabolites in *i*JO1366 with two mandatory CO_2_ assimilation reactions increases to five and 91 with glucose and glycerol as substrate.

### Thermodynamic feasibility of CO_2_-fixing pathways in ECC2

The results presented so far considered exclusively stoichiometric constraints and did not yet account for thermodynamics. Therefore, in the following we will use the concept of Max-min Driving Force (MDF; see Methods) to identify synthesis routes that are feasible with respect to both stoichiometric and thermodynamic constraints. For a given pathway, the MDF quantifies the maximal (best-case) thermodynamic driving force based on standard Gibbs free energy changes and metabolite concentration ranges. In our application, the goal was to find pathways with CO_2_ net fixation for substrate-product combinations where the MDF is greater than zero thus indicating principle feasibility of the respective pathway.

As described in the Methods section, in the core model ECC2 we computed for each substrate-product combination the set of elementary modes (EMs) and identified from this set all (stoichiometrically feasible) pathways with CO_2_ net fixation (CO_2_ assimilation yield $Y_{{CO}_{2}/C_{S}}$ larger than 0.01). By definition, for each product, the maximum CO_2_ net fixation previously calculated with FBA (Table 2) is achieved by at least one EM. For each EM with CO_2_ net fixation we calculated its respective MDF to test for thermodynamic feasibility (MDF > 0). Having the complete set of EMs at hand, we can also easily identify the EM(s) with the maximal MDF.

For glucose, 16 of the 18 identified products with stoichiometric CO_2_ assimilation in ECC2 are, in principle, thermodynamically feasible because there exists at least one EM for these metabolites with a positive driving force (Tables 2, 5, S1 and S2). In fact, we found a positive MDF for all EMs of all of these 16 products. In contrast, the previously identified metabolites asparagine and oritidine-5-phosphate must be excluded as products since the highest MDF values of their EMs for these two products are negative (-1.0, cf. Table 2). With glycerol as substrate, MDF analysis of the EMs revealed that 29 from the set of 62 products (47%) with stoichiometric CO_2_ assimilation are also thermodynamically feasible, while for 33 (53%) no EM with positive MDF could be found. For the 29 feasible products, on average about the half (48.2%) of the corresponding EMs have positive MDF values (ranging from 4.1% for succinate to 98.7% for uracil) while others are thermodynamically infeasible. The complete list of all possible substrate-product combinations together with their stoichiometric and thermodynamic properties is given in S1 and S2 Tables.

**Table 5:** Number of products in the ECC2 and *i*JO1366 model with thermodynamically feasible net CO_2_ assimilation with glucose and glycerol as substrate. For each model and substrate, the number of stoichiometrically and thermodynamically feasible substrate-product combinations with net CO_2_ fixation is given and compared with the number of all stoichiometrically feasible substrate-product combinations (in parentheses: relative proportion of thermodynamically feasible substrate-product combinations).

Model	Glucose	Glycerol
ECC2	16 of 18 (89%)	29 of 62 (47%)
*i*JO1366	34 of 41 (83%)	145 of 253 (57%)

The largest MDF for any product with CO_2_ fixation on both substrates is given by 8.6 kJ/mol (Table 2). It can be achieved with different products (e.g., orotate and the C4-metabolites of the TCA cycle) using either glucose or glycerol as substrate. Optimal MDF values were always observed at suboptimal product yields for all substrate product combinations. With glycerol as substrate, only nine of the 26 in principle thermodynamically feasible products are also feasible with maximal stoichiometric CO_2_ assimilation yield. As already indicated above, with glucose as substrate, all yield-optimal pathways to the 16 products are also thermodynamically feasible, however, always with reduced MDF compared to the maximal MDF achievable with some minimum CO_2_ fixation (Table 2). It has been suggested that an MDF of 3.0 kJ/mol would allow for large (net) fluxes [29] and we found that such an MDF can be achieved for all substrate-product combinations that are in principle thermodynamically feasible. For the majority of products this threshold can be reached either at yield-optimality or at least with only slightly reduced product yields (Table 2).

**Fig 2:**
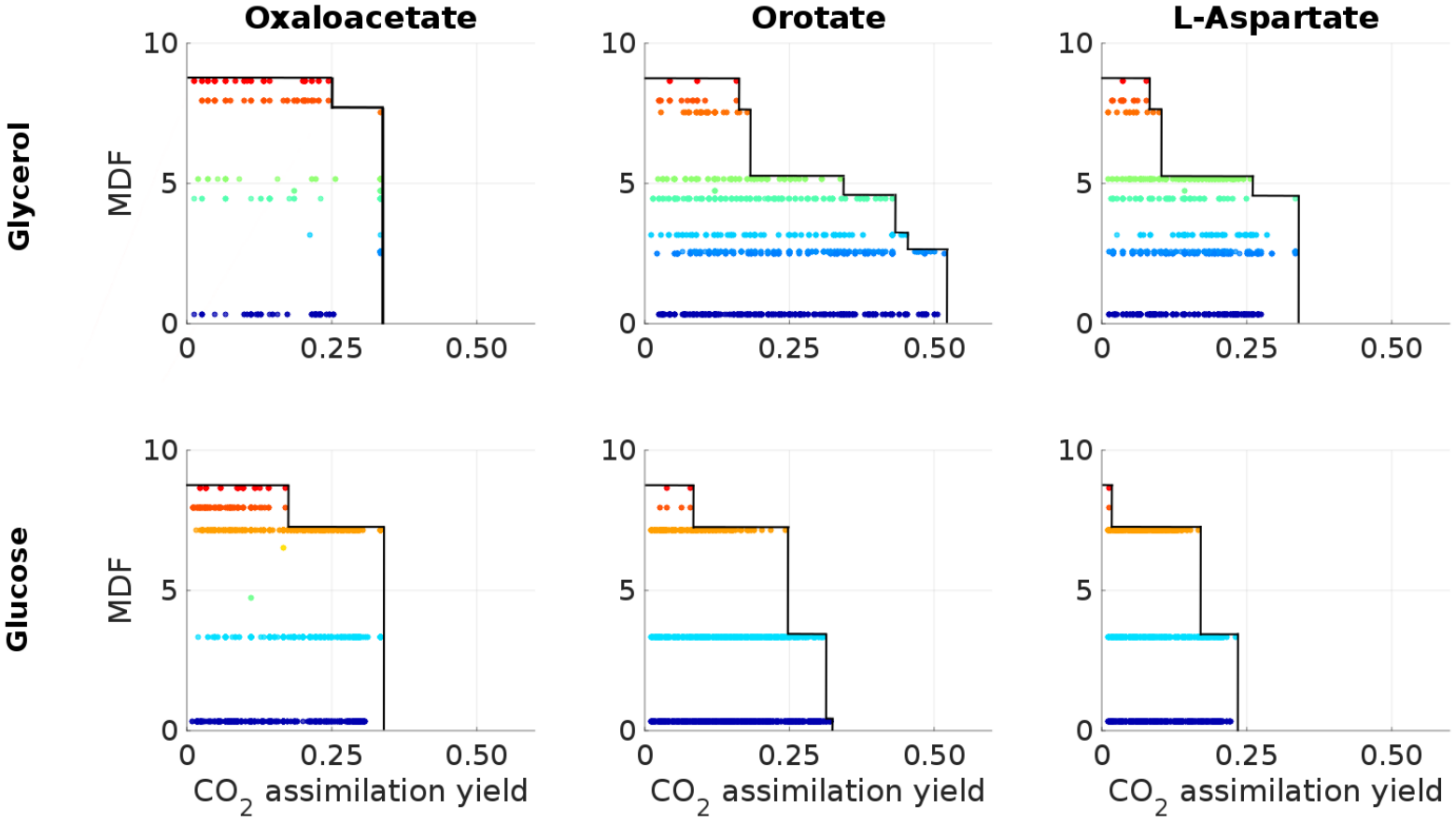
MDF and CO_2_ assimilation yield $Y_{{CO}_{2}/C_{S}}$ of EMs for selected substrate-product combinations. EMs with the same color share the same MDF. The black solid line indicates the optimal MDF for a given CO_2_ assimilation yield.

For six promising substrate-product combinations in ECC2 we investigated the relationship between MDF and CO_2_ assimilation yields in more detail (Fig 2). Although maximal MDF values usually occur at suboptimal product yields, no clear functional relationship or trend between product or CO_2_ assimilation yield and pathway MDF can be described. For specific yields there may exist broad ranges for the MDF values of the corresponding EMs. The opposite also holds true, a specific MDF value relates to many EMs that may span a wide range of possible CO_2_ assimilation yields. The most outstanding metabolites are oxaloacetate and orotate. Oxaloacetate allows for the highest carbon assimilation yield at MDF optimal conditions with both substrates. Even at maximal CO_2_ assimilation the corresponding maximal MDF values are as high as 7.5 kJ/mol and 7.1 kJ/mol, respectively. Orotate is another metabolite showing not only a high maximal MDF value but also a high carbon assimilation yield at MDF optimality.

We finally analyzed also the number of required reaction steps for synthesizing the respective products with net CO_2_ fixation (pathway lengths in Table 2) and found that the minimal number of cytosolic enzyme-catalyzed reactions of the feasible pathways within the top candidates is relatively low ranging from ten for producing oxaloacetate with glycerol to 29 for synthesizing dihydroorotate from glucose. On average, for 84.7% of these reactions thermodynamic information was available. Therefore, only a minor fraction of the involved reactions may further reduce the corresponding MDF.

### Thermodynamic feasibility of CO_2_-fixing pathways in the genome-scale model

Since MDF analysis via exhaustive enumeration and analysis of EMs as performed in ECC2 is not possible in this large-scale model, we used our new *OptMDFpathway* algorithm (Methods) to identify, for a desired phenotypic behavior (here: CO_2_ net fixation with a certain minimum yield for a given substrate-product combination), both the optimal MDF and a pathway that enables this MDF.

In *i*JO1366, 34 of the 41 stoichiometrically identified products with glucose as substrate still allow for carbon fixation if the thermodynamic constraints are taken into account. With glycerol 145 out of 253 are thermodynamically feasible (Tables 3, 5, S3 and S4). As in ECC2, maximal MDF values can always be observed at suboptimal product yields for all substrate-product combinations and the largest MDF for any product on both substrates is given by 8.6 kJ/mol which can be achieved with different products using either glucose or glycerol as substrate (Table 3). Oxaloacetate and orotate can again be identified as the most promising candidate products for both substrates (the pathway from glycerol to orotate is exemplarily shown in S10 Table). However, compared to ECC2, the increased maximal CO_2_ assimilation yields observed for some products are often accompanied with smaller corresponding MDF values resulting in even negative MDF (thermodynamically infeasible) when glycerol is applied as substrate (Table 3). However, the majority of products can at least be produced with nearly optimal product yields via pathways supporting a MDF of 3.0 kJ/mol or higher (Table 3).

**Fig 3:**
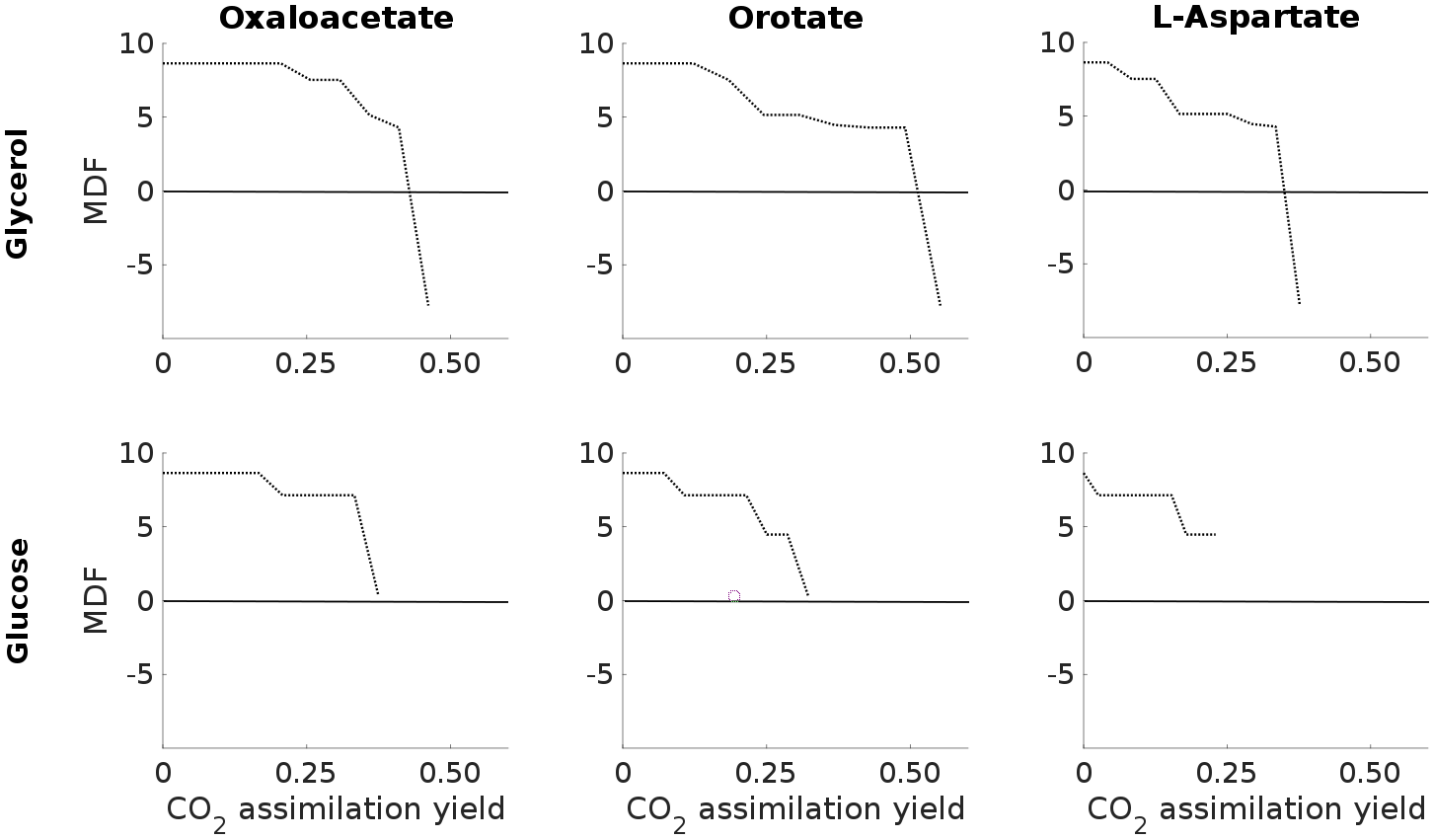
Optimal MDF depending on CO_2_ assimilation yield $Y_{{CO}_{2}/C_{S}}$ for selected substrate-product combinations in the genome-scale model *i*JO1366.

As for ECC2, we analyzed the relationships between product yields and the corresponding maximal MDF in more detail for the three promising candidates, orotate, oxaloacetate, and aspartate (Fig 3). For each of the six considered substrate-product combinations we iteratively increased the minimal carbon assimilation yield $Y_{{CO}_{2}/C_{S}}$ in discrete steps from 0 up to its corresponding maximum and computed the respective maximal MDF at each step. Since the space of flux distributions is reduced with higher CO_2_ assimilation yields, the MDF may either remain constant or will decrease with increasing product yields. As already mentioned above, in case of glycerol the very high product yields are not supported by thermodynamically feasible pathways. However, with slightly suboptimal yields considerably large driving forces are possible. In general, the behavior of the optimal MDF with respect to the CO_2_-assimialtion yield is similar to ECC2 (Figs 2 and 3) except for the higher maximal yield for oxaloacetate.

Compared to ECC2, we see that the minimal pathway lengths for the respective products are slightly shorter (Table 3). There were 13 substrate-product combinations within the set of top candidates that require less than 17 cytosolic enzyme-catalyzed reactions, the number of enzymes required for the cell-free CETCH cycle [37]. Even if we account for potential transport and exchange reactions, these numbers appear still realistic for biotechnological applications with a cell-free approach. Again, within the identified shortest pathways, for the vast majority (87.6%) of all reactions thermodynamic information was available.

**Fig 4:**
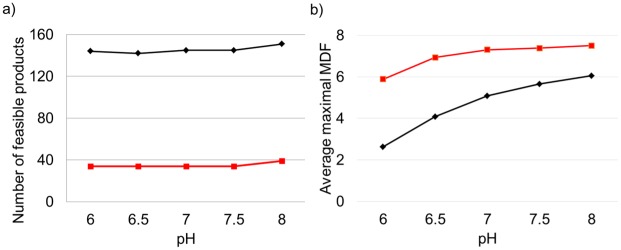
Robustness of thermodynamic feasibility of CO_2_ net fixation with respect to varying pH values. (a) Number of thermodynamically feasible products in *i*JO1366 with CO_2_ net assimilation. (b) Average maximal MDF for their synthesis. Black lines and diamonds: substrate glycerol; red line: substrate glucose.

In the work [29], Noor et al. pointed out that the MDF can be sensitive against the pH values used for the calculation of the standard Gibbs free energy change. We therefore repeated the calculations for pH-values ranging from 6 to 8 (with a step size of 0.5). We found that the results are fairly robust (see Fig 4 and S6–S9 Tables). For example the number of thermodynamically feasible products in *i*JO1366 with CO_2_ net fixation (Fig 4a) changes only slightly as there are only four metabolites (three for glycerol and one for glucose) that are feasible at pH 7 but become infeasible at other pH conditions. The opposite holds true for 13 metabolites (five for glucose and eight for glycerol) for which no thermodynamically feasible pathway exists at pH 7 but at least for one alternative considered pH condition (see S6–S9 Tables). The average maximal MDF values (for all products feasible over the whole pH range) slightly decrease for acidic pH values and increase for basic conditions (Fig 4b). However, this relationship is not generally valid for all metabolites as there are products for which the corresponding average maximal MDF decreases with increasing pH values (e.g. aspartate or oxaloacetate) or is maximal at pH 7 (e.g. orotate).

### Thermodynamic bottlenecks

The distribution of MDF values over all EMs in ECC2 shows that there are only few distinct MDF values (Fig 2). Likewise, only a limited number of different optimal MDF-values were identified in *i*JO1366 (Fig 3). This suggests that there exist a finite number of distinct thermodynamic bottlenecks (TBs) which set upper bounds for the maximal possible thermodynamic driving force. The notion of thermodynamic bottlenecks was originally introduced in [23] to mark single reactions (*localized TBs*) or groups of reactions (*distributed TBs*) that render a flux along a given pathway thermodynamically infeasible, i.e., where, from a given range of possible metabolite concentrations, no concentration vector can be found such that the driving force of each reaction *i* is positive (${{-\Delta }_{r}G}_{i}^{'}>0$). In our application we demand instead ${{-\Delta }_{r}G}_{i}^{'}>B$ but the notion of localized and distributed TBs can be directly adopted. Accordingly, a localized TB occurs if a single reaction is limiting the maximal pathway MDF because it is hitting the upper boundary value for its driving force (for reversible reactions in the respective forward or backward direction). For a localized TB, the concentrations of *all* reactants of the respective reaction reach their maximum value and *all* products their minimum value (since otherwise the MDF could be further improved). In contrast, in a distributed TB, several reactions constrain the pathway MDF simultaneously and the driving force of one involved reaction cannot be increased without lowering the driving force of another because the reactions share some metabolites. The concentration of these metabolites must be balanced such that the minimal driving force for all participating reactions is optimized.

In [29], thermodynamic bottlenecks hindering a higher MDF in a *given* pathway were identified by shadow prices of the linear MDF optimization problem. However, since *OptMDFpathway* is a MILP problem (operating on a network with possibly multiple optimal pathways with maximal MDF) this shadow price approach cannot be applied here. We therefore proceed as follows. The value of the MDF-variable *B* in Eq (8) is fixed to the previously calculated maximal MDF. With this background each driving force (*f*_*i*_*)* is separately maximized as objective in Eq (9) (instead of *B*). The reaction(s) whose determined maximal *f*_*i*_ equal the maximal MDF value previously determined for the pathway are the limiting reaction(s) forming the localized or distributed TB.

One example of a localized TB occurring in the core as well as in the genome-scale model is given by the adenylate kinase reaction (R_ADK1) which sets an upper MDF limit of 8.6 kJ/mol. Hence, the maximal MDF of all EMs in ECC2 or pathways in *i*JO1366 that comprise adenylate kinase in forward direction is given by this limit (unless an even more stringent constraint further reduces the pathway MDF). The upper limit for the driving force of adenylate kinase is hit at maximal concentrations for AMP and ATP and minimal concentration for ADP (thereby accounting for the used concentration ratio constraint of 10:1 for [ATP]:[ADP]). Another localized TB occurring in many MDF-optimal pathways with glycerol as substrate is given by the glycerol dehydrogenase reaction (R_GLYCDx) with its driving force upper limit of 7.5 kJ/mol. This limit is reached if the concentrations of cytosolic glycerol and NAD are at their respective maximal values and the concentrations of dihydroxyacetone and NADH at their minimal values. Finally, the localized TB with the smallest positive MDF is given by the malate dehydrogenase reaction (R_MDH) confirming that this reaction is a potential bottleneck for biomass or, as in our application, for product synthesis (cf. with [29]).

An example of a *distributed TB* in ECC2 for products with CO_2_ fixation with glucose as substrate is composed of the three glycolytic reactions R_TPI, R_GAPD, and R_FBA catalyzed by triose-phosphate isomerase, glyceraldehyde-3-phosphate dehydrogenase, and fructose-bisphosphate aldolase. If these three reactions, which were also identified in [29] as a thermodynamic bottleneck for glycolysis, are simultaneously used in a pathway, then the MDF is limited by 3.3 kJ/mol (again, occurrence of other bottlenecks in the same pathway may further reduce the maximal MDF). This limit is caused by the shared metabolite glyeraldehyde-3-phosphate (M_g3p_c) which is a product of reactions R_TPI and R_FBA but a substrate of reaction R_GAPD. To achieve high driving forces for R_TPI and R_FBA, low concentrations of glyeraldehyde-3-phosphate are beneficial. Contrary, sufficient driving forces for R_GAPD can only be achieved with high concentrations of glyeraldehyde-3-phosphate. Therefore, the concentration of glyeraldehyde-3-phosphate needs to be carefully balanced to enable high driving forces for all three reactions simultaneously. Altering the (optimal) concentration of M_g3p_c would increase the driving force of (at least) one reaction but lower the driving force of another thereby reducing the overall pathway driving force.

The optimal MDF values of all 62 substrate-product combinations of ECC2 are limited by only 17 different bottlenecks where the largest distributed TB limit was composed of nine reactions (S1 and S2 Tables). Over all EMs of all substrate product combinations, there were only 27 different MDF values, thereof 9 with glucose and 23 with glycerol as substrate, respectively.

The most abundant *distributed TB* with two reactions in *i*JO1366 is given by the simultaneous operation of triose-phosphate isomerase (R_TPI) together with glyceraldehyde-3-phosphate dehydrogenase (R_GAPD). If both reactions occur together in a pathway, the MDF of all routes comprising these reactions cannot be higher than 4.5 kJ/mol because glyeraldehyde-3-phosphate is a substrate of R_TPI but a product of R_GAPD. Therefore, its concentration needs to be balanced such that both reactions simultaneously achieve the highest possible driving force. Notably, this distributed TB allows for a higher driving force compared to the three-reaction TB discussed above for ECC2 which contained additionally R_FBA and caused a lower MDF limit of 3.3 kJ/mol. In *i*JO1366 alternative reactions with favorable thermodynamic properties can substitute for R_FBA making it dispensable and allowing the higher MDF value.

### Role of pyruvate synthase in iJO1366

Setting the reaction of the pyruvate synthase (R_POR5) reversible in *i*JO1366 (thus allowing activity in direction of carboxylation) increases the number of products with CO_2_ assimilation only slightly. However, for some products the maximal CO_2_ assimilation yield increases significantly. Formate is one such example: with R_POR5 being reversible, significantly higher product yields of 10.5 mol/mol glucose and 6.1 mol/mol glycerol (compared to 6.14 and 3.38 in *i*JO1366 with R_POR5 being irreversible) with accompanying molar carbon assimilation yields ($Y_{{CO}_{2}/C_{S}}$) of 0.75 and even 1.03 (compared to 0.02 and 0.13), respectively. However, as the reversibility of the endogenous R_POR5 reaction remains highly suspicious, a heterologous expression from an organism where this enzyme has been demonstrated to be fully functional in direction of carboxylation seems to be more promising. *Desulfovibrio africanus* is one possible organism whose respective enzyme showed high stability even under aerobic conditions. However, the corresponding cofactor ferrodoxin possibly needs to be transferred as well since *E*. *coli* does not possess cofactors whose redox potentials are sufficiently low.

### Biomass synthesis of *E. coli* with CO_2_ net fixation

Not surprisingly, both metabolic models used herein predict that biomass synthesis with net CO_2_ fixation. is not possible in *E*. *coli*. However, a much less intuitive result found in the genome-scale is the following: if an electron source is provided that can permanently reduce NAD^+^ to NADH (e.g., via bioelectrochemical approaches [56]), then *E*. *coli* could grow with CO_2_ as the only carbon source. Clearly, under these assumptions, ATP can be produced via respiration with NADH as electron donor but this also implies that the genome-scale model must contain a cycle with net CO_2_ fixation. A closer look revealed that such a cycle indeed exists in the model which involves reactions of the PEP carboxylase, the TCA cycle and glyoxylate shunt as well as reactions in the serine and threonine metabolism. This cycle can produce pyruvate from CO_2_ and NADH (from which then biomass can be synthesized). It has an MDF of 8.6 kJ/mol and is thus theoretically thermodynamically feasible, however, whether it would really have sufficient capacities to allow growth of *E*. *coli* solely from CO_2_ and a source of reduction equivalents remains to be shown.

### There is always an EM with maximal MDF

We finally note here an interesting theoretical result highlighting the relationship between the steady-state flux vectors with maximal MDF and the EMs (which are steady-state flux vectors with minimal (irreducible) sets of active reactions). In the smaller network ECC2, where the EMs could be enumerated, we implicitly assumed that MDF-optimal pathways with desired properties can be directly identified from the set of computed EMs. Indeed, also for genome-scale networks, it can be shown that there exists always at least one EM with maximum MDF value. The reasoning is as follows: Consider we know all EMs; this allows us to select the one with optimal MDF value. Adding further (active) reactions to this EM would impose further thermodynamic constraints, hence, the MDF either reduces or, in the best case, remains constant. Since removing a reaction from an EM implies that only the zero vector remains as feasible steady-state flux distribution, no flux vector in the network can exist with higher MDF. This implies that a solution found by *OptMDFpathway* is either an EM or it uses a superset of reactions active in an MDF-optimal EM. In the latter case, the reaction set being active in the found optimal flux vector can always be reduced to the pathway represented by the optimal EM (where the active reversible reactions in the EM are used in the same direction as in the flux vector). This is a direct consequence of the fact that every flux vector can be written as a conformal sum (sum without cancellations) of EMs [57]. If inhomogeneous constraints are used in the model definition, the same reasoning implies that there is always an elementary flux vector (a generalization of EMs [9]) with optimal MDF. These findings complement other theoretical results regarding the role of EMs for the identification of optimal pathways with respect to different objectives, for example, yield-optimal pathways [55] and pathways with maximal specific rates in kinetic models [58,59].

## Discussion

It is usually assumed that heterotrophic organisms like *E*. *coli* cannot be applied to assimilate significant amounts of CO_2_. We argue that the potential of CO_2_ assimilation by endogenous pathways of heterotrophic organisms may have been a so far overlooked component for sustainable bioprocesses consuming CO_2_. To verify this hypothesis, we systematically identified, for the first time, all combinations of two industrially important substrates and cytosolic carbon metabolites (products) in *E*. *coli* which lead to net CO_2_ fixation. By using a new optimization approach, *OptMDFpathway*, we ensured not only stoichiometric but also thermodynamic feasibility of the identified pathways.

Our analyses complement autotrophic approaches for biotechnological CO_2_ assimilation by investigating CO_2_ fixation routes that require the constant supply of carbon substrates to which CO_2_ can be attached. We demonstrated that *E*. *coli* can assimilate CO_2_ into many different metabolites: in principle, 15.3% of all cytosolic carbon metabolites in the *E*. *coli* genome-scale model can be synthesized with concomitant CO_2_ fixation from the considered substrates via thermodynamically feasible pathways. The potential products include the expected metabolites of the left branch of the TCA cycle but also less obvious candidates. We found that 40% (150 of 374 cases; Table 5) of all substrate-product combinations with stoichiometric net CO_2_ assimilation in the two networks are thermodynamically infeasible emphasizing the need of a method such as *OptMDFpathway* to filter the high percentage of thermodynamically infeasible pathways. If glucose is used as substrate, fewer products allow for net-carbon assimilation but the relative proportion of thermodynamically feasible substrate-product combinations is higher compared to glycerol (Table 5). Although the ordering and maximal carbon assimilation yields of the top-candidates remained largely unchanged, the number of possible products more than tripled when the genome-scale model *i*JO1366 instead of the core model ECC2 is used and some of the maximal product yields increase more than 10%.

The best substrate-product combination showing a high CO_2_ assimilation yield together with sufficient driving force and a small number of participating reactions was determined to be the synthesis of oxaloacetate from glycerol via glycolysis and PEP carboxylase. The optimal corresponding pathway converts one mol of glycerol into one mol of oxaloacetate, fixes one mol of CO_2_ and enables thus a molar carbon fixation yield of 0.33. The found pathway requires only one carboxylation reaction (PEP carboxylase) and supports a high MDF of 7.5 kJ/mol. Further, the pathway comprises only 13 enzyme catalyzed reactions whereof four are needed for NADH regeneration. Opposed to the proposed cell-free CETCH-cycle [37], this pathway (as all pathways identified herein) is even balanced with respect to ATP and redox cofactors such that no additional redox/energy sources must be provided. In a cell-free setup, redox cofactor regeneration could be facilitated by a bioelectrochemical transfer (by means of suitable mediators) of the electrons to an electrode. The proposed process would on the one hand further reduce the number of required enzymes (to nine) and on the other hand generate an electron flow that can be harvested to improve the overall process performance. Another interesting compound not mentioned so far which can be synthesized with a relatively high CO_2_ assimilation yield and with the highest molar product yield of all candidates is carbamoyl phosphate (Table 3). Carbamoyl phosphate is an industrially relevant product as it can be further processed to synthesize, for example, different cyanates from which insecticides or polyurethanes can be derived. In *E*. *coli* it is produced by the carbamoyl phosphate synthase (Table 1) and functions as an intermediary metabolite for nitrogen disposal through the urea cycle and for the synthesis of pyrimidines. However, when considering it as potential sink for CO_2_, a nitrogen source needs to be supplied.

Clearly, the predicted thermodynamic feasibility of the identified pathways represent best-case scenarios. Whether the upper limits of the corresponding pathway driving forces can be experimentally established *in vivo* remains to be shown. Since we restricted the MDF maximization on the set of reactions that proceed exclusively in the cytosol, it is not *per se* guaranteed that the here identified pathways can be readily applied *in vivo*. For experimental validation of particular substrate product combinations membrane transportation costs should be carefully analyzed with respect to the specific envisioned environmental conditions. Herein we did not explicitly consider introduction of heterologous enzymes and pathways in *E*. *coli* but identified the pyruvate synthase as one target for improved CO_2_ assimilation capabilities. Furthermore, apart from monetary values of substrates and products, the economic viability and overall CO_2_ sequestration yield of a process where *E*. *coli* synthesizes one of the identified products with net CO_2_ assimilation will require a more detailed analysis. For example, the CO_2_ sequestration during (photo)synthesis of the respective carbon feedstock as well as the possible CO_2_ release for growth of *E*. *coli* must then be taken into account.

The assessment of the thermodynamic feasibility of pathways with net CO_2_ fixation in the genome-scale model of *E*. *coli* was only possible with the development of the new *OptMDFpathway* method. This MILP-based algorithm determines, for a given (e.g., desired) phenotype both the optimal MDF and a supporting pathway. Herein we used this approach to assess the thermodynamic feasibility of pathways in *E*. *coli* that allow for CO_2_ net fixation but we envision that it can as well be applied to many more general problems in metabolic network modeling and design. In particular, it can be used as a generic method to identify, in large-scale networks, pathways with desired properties (e.g., synthesis of a chemical with some minimum yield) and with maximal possible driving forces.

## Supporting information

S1 Table Complete results for ECC2 with glycerol. (XLSX)

S2 Table Complete results for ECC2 with glucose. (XLSX)

S3 Table Complete results for iJO1366 with glycerol. (XLSX)

S4 Table Complete results for iJO1366 with glucose. (XLSX)

S5 Table Thermodynamic data used in the calculations. (XLSX)

S6 Table Results for pH sensitivity for ECC2 with glycerol. (XLSX)

S7 Table Results for pH sensitivity for ECC2 with glucose. (XLSX)

S8 Table Results for pH sensitivity for iJO1366 with glycerol. (XLSX)

S9 Table Results for pH sensitivity for iJO1366 with glucose. (XLSX)

S10 Table Orotate synthesis pathway with CO_2_ fixation in iJO1366. (XLSX)
